# Metabolic Adaptations of Uropathogenic *E. coli* in the Urinary Tract

**DOI:** 10.3389/fcimb.2017.00241

**Published:** 2017-06-08

**Authors:** Riti Mann, Daniel G. Mediati, Iain G. Duggin, Elizabeth J. Harry, Amy L. Bottomley

**Affiliations:** Faculty of Science, The iThree Institute, University of Technology SydneyUltimo, NSW, Australia

**Keywords:** uropathogenic *E. coli*, UPEC, urinary tract infections, metabolism, metabolomics, virulence

## Abstract

*Escherichia coli* ordinarily resides in the lower gastrointestinal tract in humans, but some strains, known as Uropathogenic *E. coli* (UPEC), are also adapted to the relatively harsh environment of the urinary tract. Infections of the urine, bladder and kidneys by UPEC may lead to potentially fatal bloodstream infections. To survive this range of conditions, UPEC strains must have broad and flexible metabolic capabilities and efficiently utilize scarce essential nutrients. Whole-organism (or “omics”) methods have recently provided significant advances in our understanding of the importance of metabolic adaptation in the success of UPECs. Here we describe the nutritional and metabolic requirements for UPEC infection in these environments, and focus on particular metabolic responses and adaptations of UPEC that appear to be essential for survival in the urinary tract.

## Introduction

Urinary tract infections (UTIs) are one of the most common bacterial infections worldwide, accounting for ~150 million cases annually (Stamm and Norrby, 2001). This places a significant financial burden on the health-care system, costing up to USD$6 billion on treatment and management each year. UTIs can be community-acquired or health-care related, and occurs in both men and women. Women are significantly more at risk with almost 50% of all women experiencing a UTI at least once in their lifetime (Foxman, 2003; Brumbaugh et al., 2013).

UTIs are categorized as complicated or uncomplicated on the basis of the type and duration of antimicrobial therapy given to the patient (Hooton, 2012; Flores-Mireles et al., 2015). Uncomplicated UTIs typically affect children, women and the elderly who are otherwise healthy and have no structural urinary tract abnormalities. These are differentiated into upper and lower UTIs, most commonly pyelonephritis (kidney) and cystitis (bladder), respectively. Uncomplicated UTIs rarely cause any serious damage, and can be naturally cleared by the host immune response even without antibiotic therapy (Hooton, 2012). Complicated UTIs on the other hand require a prolonged therapy and are associated with an increased risk of chronic and/or recurrent infection. Complicated UTIs are associated with urinary tract abnormalities such as urinary obstruction and retention, immunosuppression, previous antibiotic exposure, and renal failure. Together these factors compromise the urinary tract and increase the risk of serious complications and treatment failure (Lichtenberger and Hooton, 2008).

A range of pathogens have been implicated in UTIs, including Gram-positive and Gram-negative bacteria such as *Escherichia coli, Proteus mirabilis, Klebsiella pneumoniae, Staphylococcus saprophyticus*, and *Enterococcus faecalis*. However, the disease is predominantly caused by Uropathogenic *E. coli* (UPEC; Schulz, 2011; Flores-Mireles et al., 2015; Su et al., 2016b), which accounts for up to 75% of all cases (Foxman, 2002, 2003; Martinez and Hultgren, 2002) and 95% of community-acquired cases (Kucheria et al., 2005).

UPEC are a pathotype of extraintestinal pathogenic *E. coli* (ExPEC) and originate from the intestinal microbiome. Within the intestine, UPEC rarely cause any complications and exist in a beneficial symbiotic relationship with intestinal microflora (Wiles et al., 2008). However, UPEC have adapted the ability to disseminate and colonize other host environments such as the urinary tract and bloodstream. Virulence factors, such as toxins, modify, and damage the host to promote infection (Flores-Mireles et al., 2015). In addition, physiological factors that do not directly damage the host but nevertheless are essential for UPEC growth and survival in the urinary tract are now being appreciated for their role in pathogenesis (Alteri and Mobley, 2012). The capacity of UPEC to utilize nutritionally-diverse environments such as the intestines, urine, bladder, kidneys, and bloodstream clearly plays a significant role in its pathogenesis (Brown et al., 2008; Eisenreich et al., 2010); UPEC metabolism is tightly regulated and highly responsive to nutrient availability, allowing survival with a wide range of nutrients in competitive, fluctuating environments.

This review will discuss the nutritional and metabolic responses of UPEC as it moves from the intestine to the harsh environment of the urinary tract, where virulent infection starts. Nutrients available for UPEC growth in the intestine and urinary tract have been compared in order to explore these two environments as growth mediums for UPEC. Genetic and metabolic responses that allow UPECs to survive in and cause infection in the urinary tract are discussed in relation to the nutrients available inside the urinary tract. As the role of metabolism during UPEC infection is critical to advance our understanding of pathogenesis, we reflect on the prospect of UPEC metabolism as a potential drug target, to combat the antibiotic resistance that has already developed against 70% of the drugs currently being used clinically for UPEC-induced UTIs (Foxman, 2002).

## UPEC lifestyle from intestine to the urinary tract

The gastrointestinal tract is considered the primary reservoir of UPEC in humans. For successful colonization in their main habitat, the colon, *E. coli* first needs to survive passage through the acidic conditions of the stomach, and upper intestine, and then penetrate the viscous upper mucus layer of the colon epithelium (Møller et al., 2003), survive other host defense mechanisms (Bergstrom et al., 2012) and compete with other strains and species of the complex intestinal surface microbiota for acquisition and utilization of nutrients (Freter et al., 1983; Rang et al., 1999; Miranda et al., 2004). Some *E. coli* cells remain or are shed into the intestinal lumen and then excreted in feces.

**Figure 1:**
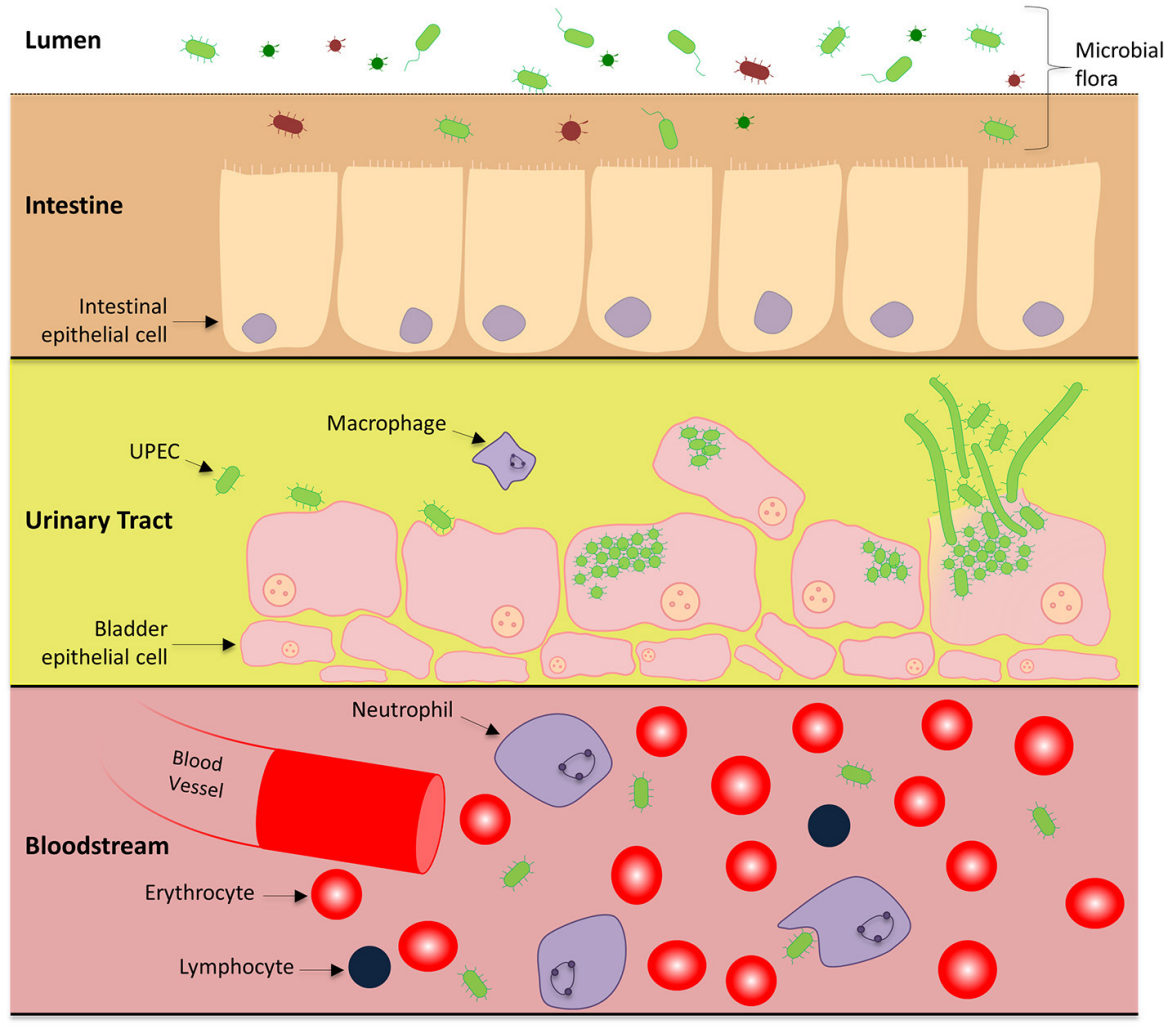
UPEC pathogenesis across multiple microenvironments: Uropathogenic *E. coli* harmlessly grow in the human intestines as part of the microbiome. Within this environment, UPEC interact with the intestinal epithelial cells in a symbiotic relationship, however there is competition for nutrients between other microorganisms. UPEC has also adapted the ability to cause urinary tract infections and urosepsis by transitioning to a pathogenic lifecycle in the urinary tract and bloodstream. To gain a stronghold within the urinary tract, UPEC express numerous pili systems to facilitate attachment to the superficial bladder epithelial cell layer. Invasion into the host cell initiates replication immediately to form IBCs and a subpopulation undergoes cell elongation (filamentation). Eventually the epithelial cell is overloaded and UPEC escape, rupturing open the host cell releasing motile short and elongated cells which can infect neighboring host epithelia to continue the infective cycle.

UTIs are usually initiated when UPEC contaminate and colonize the urethra and migrate into the bladder lumen. Most of the characterized strains of UPEC invade the bladder epithelium and undergo an intracellular infection cycle (Martinez et al., 2000; Justice et al., 2004) and there is evidence that this occurs in most human UTIs (Rosen et al., 2007). The infection cycle is a complex pathway involving epithelial attachment, invasion of host cells, and intracellular proliferation, leading to the eventual rupture of the bladder epithelial cell, dissemination and reinfection of surrounding epithelial cells (Justice et al., 2004, 2006a; Andersen et al., 2012). Infections of the lower urinary tract have the potential to progress to the kidneys and enter the bloodstream causing potentially fatal urosepsis, as shown in Figure 1 (Flores-Mireles et al., 2015).

Many studies have focused on virulence factors that play a role in mediating UPEC colonization of the bladder, most notably the initial attachment of bacterial adhesive structures to the luminal surface of the bladder epithelium. This step of infection has been quite well characterized and has been a focus of attention in the development of therapies for UTIs (Langermann et al., 2000; Asadi et al., 2014). UPEC surface fibers, in particular Type 1 pili and their tip adhesin (FimH), recognize mannosylated glycoprotein receptors present on the host epithelium, mediating UPEC binding (Jones et al., 1995; Martinez et al., 2000). UPEC also express a variety of other proteins on their surface, which facilitates bacterial attachment (see Mulvey, 2002). Once bound, UPEC may become internalized via endocytosis (Mulvey et al., 1998); activation of the host Rho-family proteins, such as RhoA, Cdc42, and Rac1 result in the rearrangement of the host cell membrane, engulfing the bacteria in endocytic vesicles (Martinez et al., 2000; Martinez and Hultgren, 2002).

Within the intracellular environment, which offers a degree of protection from innate immune responses, UPEC grow as dense clusters of bacteria encased in a polysaccharide-rich matrix (Anderson et al., 2003). These clusters, known as intracellular bacterial communities (IBCs), have been observed in the murine model and the human cell-culture model of UTI, as well as shed into human urine (Anderson et al., 2003; Justice et al., 2004; Rosen et al., 2007; Andersen et al., 2012). As UPEC proliferate and the cluster develops to overwhelm the host cell, subpopulations of differentiated bacteria develop, including highly motile bacteria and extensive bacterial filaments—cells that have continued growth with an arrest of cell division (Justice et al., 2004, 2006b; Andersen et al., 2012). The development of motile and filamentous bacteria accompanies host cell rupture, and promotes dissemination, adhesion and colonization of surrounding host surfaces, whilst helping avoid consumption by the innate immune system (Justice et al., 2006b; Andersen et al., 2012). UPEC filaments have the potential to divide and reform to the regular rod-shaped cells that are able to invade new host cells (Rosen et al., 2007; Andersen et al., 2012). During these changes, UPEC would experience rapid substantial changes in nutritional status.

## Nutritional aspects of the intestine and urinary tract environments for UPEC

UPEC must rapidly adapt to new environments as they transition between the intestinal lumen and urinary tract, in both intracellular and extracellular host microenvironments. In the urinary tract, urine is one of the major sources of nutrients encountered by UPEC during the extracellular phase of the infection cycle. Table 1 outlines the principle compounds that demonstrate the potential to serve as sources of essential elements within the intestine and urine that can support UPEC growth.

**Table 1:** Potential sources of essential elements in two of the principal environments supporting UPEC growth, the upper mucus layer in the colon, and urine in the urinary tract, with concentrations in indicated where known (C-carbon, O-oxygen, H-hydrogen, N-nitrogen, S-sulfur, P-phosphorus, K-potassium, Mg-magnesium, Ca-calcium, Fe-iron, Na-sodium, Cl-chlorine).

**Intestine**		**Urine**			
**Nutritional source**	**Elementary requirement of UPEC fulfilled**	**Nutritional source**	Concentration (Mean ±*SD*)^*^	**References**	**Elementary requirement of UPEC fulfilled**
Polysaccharides	C, O, H	Urea	22.5 ± 4.4 mM	Bouatra et al., 2013	C, N, O, H
Dietary fibers	C, O, H	Creatinine	10.4 ± 2.0 mM	Bouatra et al., 2013	C, N, O, H
Glycoproteins (N-acetylneuraminic acid, N-acetylglucosamine)	C, N, O, H	Hippuric acid	298.5 ± 276.8 μM	Bouatra et al., 2013	C, N, O, H
Glycolipids	C, O, H	Organic acids: Citric acid	280.6 ± 115.2 μM	Bouatra et al., 2013	C, O, H
Proteins	C, N, O, H, S	Amino acids: D-serine	3.0−40 μg/mL	Cosloy and McFall, 1973; Brückner et al., 1994; Pätzold et al., 2005	C, N, O, H
Lipids	C, O, H	Nucleic acids	–	Bouatra et al., 2013	C, N, O, H, P
Nucleic acids	C, N, O, H, P	Oxytocin	0.9 ± 0.1 pM	Bouatra et al., 2013	C, N, O, H, S
Phospholipids	C, O, H, P	Angiotensin II	1.2 ± 0.2 pM	Bouatra et al., 2013	C, N, O, H
Ribose	C, O, H	Melatonin	3.3 ± 2.7 pM	Bouatra et al., 2013	C, N, O, H
Fucose	C, O, H	Ammonia	2.8 ± 0.9 mM	Bouatra et al., 2013	N, H
Mannose	C, O, H	Sodium	14.7 ± 9 mM	Bouatra et al., 2013	Na
Gluconate	C, O, H	Chlorine	8.8 ± 6.2 mM	Bouatra et al., 2013	Cl
Other dietary elements	K, Mg, Ca, Fe	Potassium	4.6 ± 0.1 mM	Bouatra et al., 2013	K

* *Concentrations in urine are normalized to creatinine concentration, considering a constant rate of creatinine excretion for each urine sample and to correct for dilution as different volumes of urine samples were taken for analysis (Bouatra et al., 2013)*.

### Intestine

Within the intestine, most UPEC strains are thought to exist like other strains of commensal *E. coli*, as they do not directly cause pathogenic infection there. The major source of nutrients utilized by *E. coli* is provided by the mucus layer in which they live, which contains surface-associated and secreted glycoproteins (mucins) that are catabolized by the intestinal microbiota to yield a variety of sugars, including N-acetylneuraminic acid and N-acetylglucosamine that UPEC can utilize as a source of carbon and energy (Severi et al., 2007; Katouli, 2010). Proteins, lipids and nucleic acids present in the intestinal mucus layer, including those originating from shed host epithelial cells and exogenous dietary fiber may also be utilized as a nutrient source (Conway et al., 2004). Degraded components of mucus and the epithelium may also diffuse into the intestinal lumen, where they can be utilized during UPEC metabolism within the intestine. The competition for carbon sources, particularly sugars, in the complex ecosystem of the colon is considered the main nutritional limitation for *E. coli* in this environment (Conway and Cohen, 2015). Consequently, *E. coli* have developed the capacity to simultaneously metabolize numerous types of sugars, and independently and rapidly regulate their production of the enzymes required, in order to compete (Maltby et al., 2013). Essential salts and metal ions are present in sufficient quantities, however, some essential ions, particularly iron and zinc, may be limiting and subject to intense competition (Gielda and DiRita, 2012; Kortman et al., 2014).

### Urine

The urinary tract offers a very different and diverse set of microenvironments that may be utilized by UPEC if the numerous nutritional and immunological stresses encountered here can be overcome. UPEC nutrition and metabolic responses during the invasive intracellular infection phases are described further below. Here, we outline the constituents of urine in relation to UPEC nutrition, since survival or growth in urine is common to UPEC's successful transition between the multiple surface and sub-surface niches throughout the urinary system.

Understanding the complete metabolic profile of urine that can be utilized by UPEC is challenging mainly due to urines great compositional complexity and variability. Nonetheless, advances in techniques such as metabolomics allow large-scale simultaneous analysis of compounds in complex biological mixtures and have greatly expanded knowledge of the metabolic profile of urine (Patti et al., 2012).

#### The human urine metabolome database

All metabolites identified in pooled human urine to date are available in a database termed the Urine Metabolome Database (UMDB; Bouatra et al., 2013). Currently it contains 2,651 metabolites, with 220 metabolites specifically associated with different conditions of disease. Although, the human urine metabolome shows some similarity to the human serum metabolome (Psychogios et al., 2011), there are more than 484 compounds uniquely identified in urine (Bouatra et al., 2013). Whilst this indicates that there may be a subset of metabolites that are unique to urine, it is likely that the composition of urine is similar to blood, although certain metabolites from the blood are most likely concentrated by the kidneys, resulting in them being present at much higher concentrations compared to serum, and thus above the detection levels for current metabolome analysis (Bouatra et al., 2013).

Urine contains relatively high concentrations of urea, creatinine, amino acids, organic acids, inorganic ions such as ammonia, sodium and potassium, purines, pyrimidines, and many water-soluble toxins, which must be either utilized or tolerated by UPEC (Table 1; Vejborg et al., 2012; Bouatra et al., 2013). In fact, urine contains 5–10 times more compounds and exhibits 2–3 times more chemical diversity as compared to other biological fluids like saliva (Takeda et al., 2009) and cerebral spinal fluid (Mandal et al., 2012). The major metabolites of urine are amino acids and carbohydrates. D-serine is the most abundant amino acid in mammalian urine present at concentrations between 30 and 379 μMol/L (Brückner et al., 1994; Pätzold et al., 2005), whilst organic metabolites such as hydroxyl acid, citric acid, ammonia, creatinine, and hippuric acid are also present in significant quantities (Table 1). Despite containing high concentrations of many metabolites that may be utilized by UPEC, urine is classified as a fairly nutrient-limited growth medium, because arginine, methionine, valine, uracil, adenine and isoleucine are in limited supplies (Vejborg et al., 2012), and iron is present in limited concentrations (Stamey and Mihara, 1980), similar to many host environments harboring bacteria. UPEC would largely rely on its capacity for *de novo* synthesis of these and many other essential metabolites. The least abundant metabolites available to UPEC in urine include sugars, particularly glucose, specific peptides and hormones such as oxytocin, angiotensin, and melatonin (Bouatra et al., 2013); however despite being present in such low concentrations these components do have the potential to fulfill the elementary requirements of UPEC when catabolized. Thus, whilst urine is perhaps nutritionally more challenging to support bacterial growth compared to the intestinal environment, uropathogens, including most UPEC strains, have well-developed metabolic capacity to survive or thrive in this and other environments of the urinary tract.

## Genetic and metabolic responses in UPEC pathogenesis

The consequence of the metabolic diversity of urine on the growth of UPEC within the urinary tract still remains to be fully understood. Considering the different nutritional standings of the intestine and urinary tract and the variability within each, urinary metabolites are most likely to activate genetic and metabolic adaptive strategies in UPEC in order to survive and cause infection inside the urinary tract, some of which are detailed here.

### Central carbon metabolism

**Figure 2:**
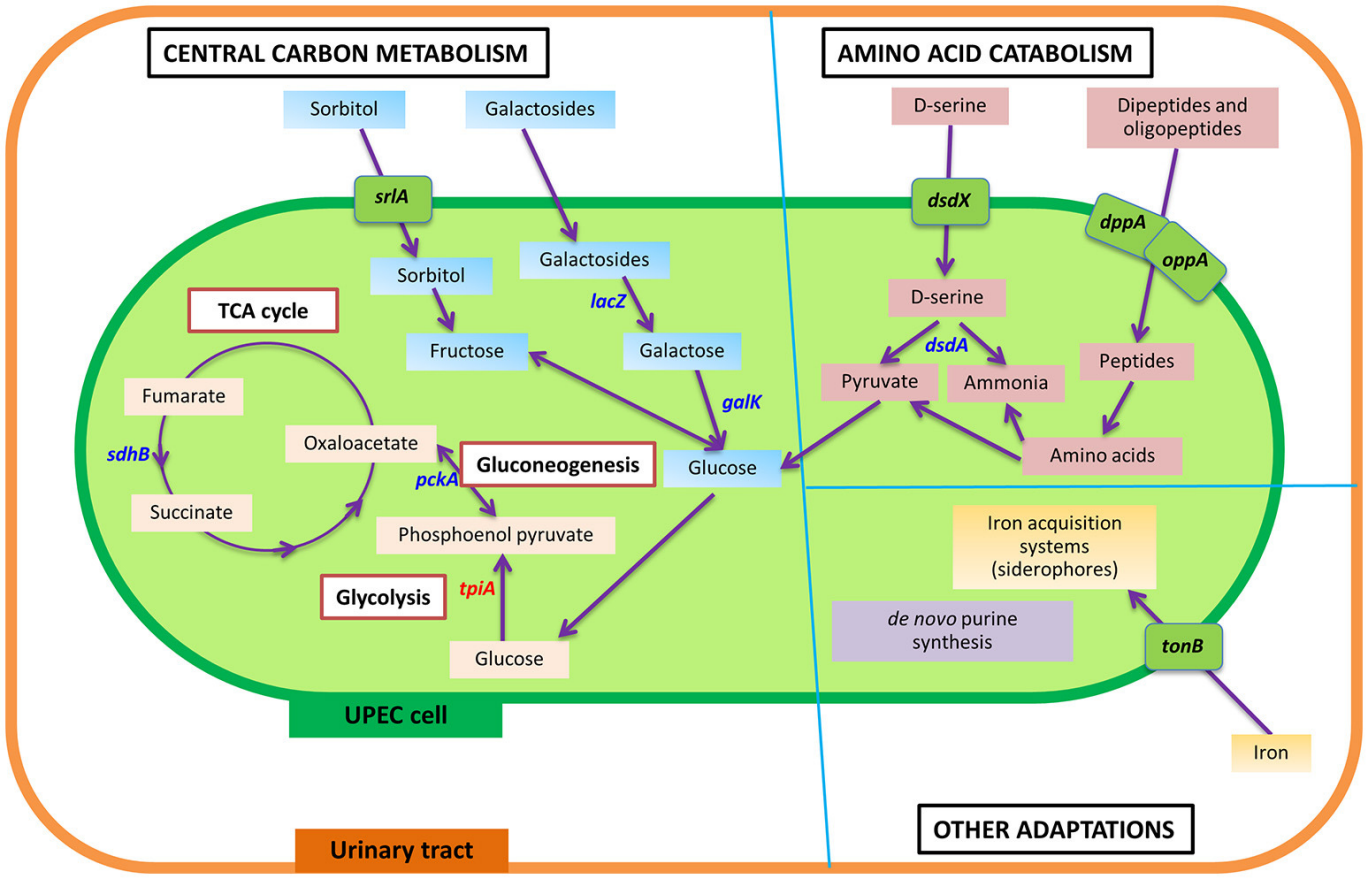
Summarized view of the main metabolic responses of UPEC during urinary tract infection, as detailed in text under the section “Genetic and metabolic responses in UPEC pathogenesis.” In comparison to the intestine, wherein glycolysis and Entner-Doudoroff pathways are shown to be important for UPEC survival, UPEC in the urinary tract displays a number of metabolic adaptations in central carbon metabolism, amino catabolism and other pathways, to cause infection in the urinary tract. Genes identified to play a role in UPEC pathogenesis are shown, having detailed information in text. Genes in blue color denote genes that play a role in UPEC fitness in the urinary tract, or result in attenuation in a mouse or *in vitro* model of invasion and intracellular bacterial community formation. The gene in red denotes a gene specifically identified to play a role in kidney infection. Transporters are shown in green boxes over the cell membrane. *sdhB*, succinate dehydrogenase; *pckA*, phosphoenolpyruvate carboxykinase; *tpiA*, triosephosphate isomerase; *srlA*, sorbitol transporter; *lacZ*, β-galactosidase; *galK*, galactokinase; *dsdX*, D-serine specific transporter; *dsdA*, D-serine deaminase; *dppA*, periplasmic dipeptide transport protein; *oppA*, periplasmic oligopeptide-binding protein; *tonB*, ferric iron uptake mediator; *pur*, genes involved in purine synthesis.

During intracellular UPEC colonization of the bladder and kidneys, aerobic respiration and carbon metabolism are essential for survival (Figure 2; Alteri et al., 2009). UPEC pyelonephritis isolate CFT073 defective in *sdhB*, the succinate dehydrogenase enzyme involved in the conversion of fumarate to succinate during the tricarboxylic acid cycle, is significantly outcompeted by the isogenic parental strain in mouse bladder and kidney UTI models (Alteri et al., 2009). Similarly, disruption of gluconeogenesis by deletion of *pckA*, encoding for phosphoenolpyruvate carboxykinase which catabolizes oxaloacetate to phosphoenol pyruvate, results in a significant reduction in fitness to colonize both bladder and kidneys *in vivo* when compared to wild-type (Alteri et al., 2009). However, mutants defective in gluconate catabolism of the Entner-Doudoroff pathway, by deletion of *edd*, do not affect *in vivo* fitness in a mouse UTI model, suggesting this pathway is dispensable in UPEC pathogenesis of UTIs (Alteri et al., 2009), and indicates a specific metabolic adaptation by UPEC during bladder infection that utilizes gluconeogenesis and the tricarboxylic acid cycle. It should be noted that the infection model used in this study only demonstrates the fitness defect in the mutants of these enzymes as compared to wild type and does not rule out the importance of other metabolic adaptations during UPEC infection. Interestingly, in a kidney mouse model of UTI, deletion of *tpiA*, the triosephosphate isomerase involved in glycolysis, was found to have attenuation and a colonization defect in the mouse kidneys, but not in the bladder (Alteri et al., 2009). Thus, it appears that the glycolysis pathway is dispensable during the initial establishment of infection within the bladder, but is critical for colonization of the kidneys. This evidence suggests that gluconeogenesis and the tricarboxylic acid cycle must come to completion for UPEC pathogenesis during intracellular infection of the bladder, whilst glycolysis is important in kidney colonization during UTIs.

β-galactosides and sorbitol appear to provide a major source of carbon during intracellular infection in the urinary tract as both the sorbitol transporter, encoded by *srlA*, and the galactoside catabolism gene, *lacZ* (encoding β-galactosidase that cleaves the glycosidic bond in galactosides to release galactose), were found to be significantly upregulated during bladder cell colonization (Conover et al., 2016). This is further supported by *galK* mutants (the gene product being involved in conversion of galactose to glucose) producing significantly smaller IBCs compared to the wild-type in a mouse UTI model, with both mutants eventually being out-competed after 2 weeks of intracellular infection (Conover et al., 2016). Positive X-Gal (5-bromo-4-chloro-3-indolyl-β-D-galactopyranoside) staining of UPEC during the IBC stage of a mouse UTI model also implies strong β-galactosidase activity (Justice et al., 2006b). Together, evidence suggests glucose availability within intracellular infection is limiting and UPEC are adapted to utilize galactose and sorbitol as their prime carbon sources. This is in accordance with urine metabolome studies, which show that glucose is rarely abundant in the urinary tract unless in a diabetic situation (Bouatra et al., 2013; Mobley, 2016).

Whilst glycolysis appears to have a critical role in the colonization of kidneys by UPEC, the importance of this metabolic pathway is not restricted to this *E. coli* pathotype or host environment. Both glycolysis and the Entner-Doudoroff pathway are vital for the survival of commensal *E. coli* in the mouse intestine as the deletion of *pgi* (phosphoglucose isomerase) and *edd* (6-phosphogluconate dehydratase) genes, having major roles within glycolysis and the Entner-Doudoroff pathway respectively, each display attenuated intestinal colonization in a competitive murine colonization model (Sweeney et al., 1996; Chang et al., 2004). Conversely, whilst gluconeogenesis and the tricarboxylic acid cycle are dispensable during UPEC intestine colonization (Chang et al., 2004), they are critical for UPEC survival during bladder infection, demonstrating the metabolic flexibility of UPEC to adapt to nutrient availability within diverse host environments.

### Amino acid catabolism

The ability of UPEC to exploit the high mixtures of amino acids and complex small peptides present within human urine has long been considered an adaptation to this environment. The utilization of these resources by UPEC is supported by the upregulation of genes involved in amino acid catabolism during growth in human urine, such as *dsdA* (D-serine deaminase) (Snyder et al., 2004), and microarray studies have demonstrated the upregulation of arginine, serine and histidine transporters in UPEC during urine growth when compared to Luria-Bertani media (Conover et al., 2016). Both serine and arginine auxotrophs do not exhibit any major fitness defects in a mouse UTI model (Alteri et al., 2009), indicating that these amino acids are effectively utilized from the growth milieu. Small peptide uptake is also important for UPEC infection, as *dppA* (periplasmic dipeptide transport protein) and *oppA* (periplasmic oligopeptide-binding protein) deletion mutants defective in peptide transport in CFT073, were significantly out-competed by wild-type in the mouse bladder model of UTI (Alteri et al., 2009). Interestingly, a subset of genes involved in amino acid utilization and synthesis, most notably tryptophan and cysteine, were downregulated during the IBC stage of infection (Conover et al., 2016), and strains that are auxotropic for these amino acids have the ability grow to a high density in urine (Hull and Hull, 1997). This suggests that short peptide and selective amino acid uptake and catabolism is necessary for growth in human urine and correlates with the high concentrations of amino acids reported in the metabolomic analysis of human urine (Table 1). However, small peptides and amino acids must be available from alternate sources apart from urine during the infection cycle, as UPEC mutants auxotrophic in arginine, glutamine and guanine synthesis have all shown a severe growth defect in human urine, while leucine, methionine and phenylalanine auxotrophs displayed a moderate growth rate (Hull and Hull, 1997).

A potential metabolic signaling mechanism evident in UPEC during urine growth is that of the amino acid D-serine, present in high concentration (3.0–40 μg/mL) in human urine (Cosloy and McFall, 1973; Brückner et al., 1994; Pätzold et al., 2005). Whilst potentially bacteriostatic at these concentrations, the expression of D-serine deaminase in UPEC isolates allows for catabolism of this metabolite (Roesch et al., 2003; Snyder et al., 2004). Because of this, catabolism of D-serine was suggested to be a vital signaling mechanism for virulence factor expression as deletion of *dsdA* (encoding D-serine deaminase) displays a prolonged log phase when grown in urine and a supposed hyper-colonization phenotype in a competitive murine UTI model (Anfora et al., 2007, 2008). Contrary to this, a subsequent study demonstrated that the *dsdA* deletion does not lead to hyper-colonization and that any gain of fitness phenotype previously reported is likely due to an unrecognized secondary mutation in the sigma factor *rpoS* gene (Hryckowian et al., 2015). RpoS associates with the core RNA polymerase to control the expression of ~10% of genes in *E. coli*, notably when under stressful nutrient-limiting conditions (Weber et al., 2005). This suggests that the unrecognized secondary mutation in *rpoS* may have affected the *dsdA* mutant. Although D-serine may play a role as a signaling mechanism for virulence factor expression, and UPEC displays adaptive measures to allow growth in the presence of this bacteriostatic metabolite, evidence suggests that D-serine does not affect the capacity of UPEC to colonize the murine urinary tract (Hryckowian et al., 2015).

### Purines and pyrimidines

UPEC can also adjust metabolic capacity to maintain growth and colonization in the human bloodstream, subsequently causing urosepsis in a small number of cases. The capacity to defy the bactericidal activity of human serum and colonize the bloodstream represents a crucial virulence advantage for UPEC. It is possible that this virulence advantage could be due to metabolic adaptation: mutagenesis studies identified genes defective in both the *de novo* purine and pyrimidine nucleotide biosynthetic pathway to be attenuated for commensal *E. coli* growth in human serum after 24 h (Samant et al., 2008). This study, making use of the Keio collection of mutants displayed the greatest growth reduction in the *purA, purE, pyrC*, and *pyrE* gene deletions when compared to growth in Luria-Bertani media (Samant et al., 2008). These defects were complimented and restored when the appropriate nucleotide base precursor was supplemented in the serum. This suggests that nucleotide sugars and precursors are limiting for growth in human serum forcing *E. coli* to undergo *de novo* biosynthesis to colonize. Whilst this metabolic adaptation has not been demonstrated for UPEC survival in serum directly, changes in the regulation of purine and pyrimidine synthesis has been observed in the cystitis UTI89 strain (Hadjifrangiskou et al., 2011; discussed below). Thus, the direct role of purine and pyrimidine synthesis for UPEC metabolic adaptation for growth in serum remains an interesting area to explore.

### Iron uptake and transport systems

UPEC express a variety of iron uptake and transport systems such as iron-chelating siderophores and hemophores during intracellular infection. Iron siderophores are low molecular weight metal chelators involved in ferric iron scavenging and play a major role in binding available iron to promote UPEC intracellular colonization within the iron-limiting urine and bladder epithelial cells. Transcriptome studies have identified various genes involved in siderophore synthesis to be dramatically upregulated during *in vivo* mouse UTI infection, most notably salmochelin and yersiniabactin (Conover et al., 2016). Further, greater production of yersiniabactin and salmochelin in UPEC as opposed to commensal *E. coli* suggest their possible role in facilitating infection of the urinary tract by UPEC (Henderson et al., 2009). Interestingly, the ferric iron uptake mediator TonB, which interacts with bacterial outer membrane receptors to facilitate iron uptake, has shown to be important in the mouse kidney model of UTI, with a deletion of *tonB* resulting in a reduction in UPEC colonization at 48 h post-infection when compared to wild type (Torres et al., 2001). This highlights the necessity of iron uptake and transport systems in UPEC pathogenesis.

The withholding of free iron by the host is usually mediated by iron-binding proteins like lactoferrin, ferritin, transferrin, ovalbumin and siderocalin (also known as lipocalin-2) (Miethke and Marahiel, 2007). In an effort to counteract bacterial iron-scavenging proteins, the host protein siderocalin, introduced into the urinary tract by leukocytes and uroepithelial cells, can restrict the accessibility of iron to UPEC by scavenging ferric iron complexes (Goetz et al., 2002). Siderocalin shows very high affinity to the bacterial siderophore enterobactin, which is found ubiquitously in *E. coli* strains. However, the lower binding affinity of siderocalin to other siderophores such as aerobactin, salmochelin, and yersinibactin which are more often associated with UPEC strains (Fischbach et al., 2006; Henderson et al., 2009), may provide an evolutionary advantage for UPEC during host iron scavenging. Indeed, purified siderocalin is capable of effectively inhibiting *E. coli* K-12 that produces only enterobactin. However, no effect was observed on the growth of *E. coli* that produces all siderophores, such as UPEC (Garénaux et al., 2013).

The composition of human urine plays a role in the regulation of siderocalin activity: an as-yet-unidentified urine constituent inhibits the host siderocalin-mediated growth restriction, allowing the bacterial siderophore enterobactin to overcome the inhibitory iron-binding effects of siderocalin and scavenge host iron (Shields-Cutler et al., 2015). Conversely, the antibacterial activity of siderocalin was found to be associated with urinary pH, with increasing pH shown to promote siderocalin activity (Shields-Cutler et al., 2015). Other the other side, acidic pH alone increases the solubility and availability of iron partly contributing to the differences in iron availability in solution and reducing the effect of siderocalin on bacterial siderophores. Metabolomics studies identified the association of increased urinary aryl sulfate metabolites, independent of the urinary pH, with the antibacterial activity of siderocalin (Shields-Cutler et al., 2015). The involvement of urinary constituents in controlling the bacterial growth points to a urinary tract-specific host-pathogen interaction system based on host metabolites and bacterial siderophore biosynthesis during UTIs (Shields-Cutler et al., 2015). This indicates that the host siderocalin uses urinary metabolites as cofactors for restricting the availability of iron to *E. coli* (Shields-Cutler et al., 2015) and that niche-specific roles of siderophores drive their diversity among UPEC (Chaturvedi et al., 2014; Shields-Cutler et al., 2015). Thus, whilst UPEC has developed adaption strategies to utilize metabolites present in host urine, these metabolites can also be utilized by the host as a defense mechanism against UPEC infection.

### Other known metabolic regulators of UPEC pathogenesis

As well as the metabolic adaptation to survive and thrive within the above environments, nutritional sensing is vital for UPEC to efficiently respond to nutrient-fluctuating conditions within the host environment, with several regulatory systems, as discussed below, reported to be important for UPEC virulence.

#### RyhB

UPEC not only requires to upregulate the genes required for iron-acquisition under iron-limited conditions of the urinary tract, but also needs to precisely maintain the levels of iron required for survival. The sRNA RyhB and the global ferric uptake regulator Fur have been implicated for their role in iron acquisition and controlling the storage of iron in *E. coli* (Massé and Gottesman, 2002). Fur and RyhB regulate the activity of cellular processes such as metabolic pathways, with TCA cycle enzymes known to be targeted by RyhB in *E. coli* (Massé et al., 2005). RyhB is important for colonization of the urinary tract by UPEC strain CFT073 via the production of iron-acquisition systems, with a Δ*ryhB* mutant producing lower levels of the siderophores aerobactin, salmochelin, and enterobactin (Porcheron et al., 2014). Therefore, RyhB is shown to play a role in virulence of the UPEC strain CFT073 (Porcheron et al., 2014).

#### BarA-UvrY

The two component system BarA-UvrY responds to nutritional fluctuations through the action of regulatory RNAs CsrB and CsrC and CsrA protein activity, which regulates expression of genes involved in gluconeogenesis and glycolysis (Romeo, 1998; Suzuki et al., 2002; Weilbacher et al., 2003). Loss of the response regulator UvrY results in decreased fitness in both monkey cystitis and murine ascending UTI competition models (Tomenius et al., 2006; Palaniyandi et al., 2012), and decreased invasion of uroepithelial cells (Palaniyandi et al., 2012). Thus, the ability to effectively sense and utilize the mixture of gluconeogenic and glycolytic carbon sources within urine is a key metabolic adaption for UPEC survival.

#### QseBC

The two-component regulatory pathway system QseBC, consists of QseC which functions as a sensory kinase to the environment stimuli and host signal, resulting in phosphorylatation of the transcription factor QseB, acting as the response regulator in UPEC. This has been demonstrated by analyzing the *qseC* mutant, which creates a positive feedback loop constantly expressing QseB (Hadjifrangiskou et al., 2011). Furthermore, deletion of *qseC* in the cystitis isolate UTI89 leads to attenuated virulence and altered expression of 36 regulators, 52% of which modulate at least 18 metabolic pathways (Hadjifrangiskou et al., 2011). These include an increase in pyrimidine production and utilization, whilst purine biosynthetic genes, *purK, purM* and *purT*, as well as arginine synthesis genes, *argF, argG*, and *argR* show downregulation (Hadjifrangiskou et al., 2011). Bypassing purine production by the addition of guanosine or adenine monophosphate was found to restore growth in a *qseC* mutant, highlighting the importance of purine metabolism for UPEC survival. This is interesting as *de novo* purine biosynthesis has been recently shown to be essential for proliferation of UPEC within the bladder during IBC, with Δ*purF* and Δ*cupA-purF* operon displaying impaired fitness at 3, 6, and 16 h post-infection *in vivo* (Shaffer et al., 2017).

#### fnr

Virulence gene expression also needs to be coordinated in response to the availability of oxygen during oxygen-limiting conditions within the bladder (Marteyn et al., 2010). Within UPEC, the transcriptional regulator *fnr* controls oxygen-related gene expression under anaerobic conditions. This regulator, which contains a DNA-binding domain, was first identified when the *fnr* mutant was no longer able to use fumarate and nitrate as substrates during anaerobic growth (Lambden and Guest, 1976), and also results in reduced metabolism of α-ketoglutarate under oxygen-limited conditions (Barbieri et al., 2014).

Evidence suggests that, in addition to acting as an aerobic-anaerobic metabolic regulator for UPEC, *fnr* controls virulence gene expression within the bladder environment (Lambden and Guest, 1976). The deletion of *fnr* within UPEC has shown a decreased adherence and subsequently invasion capacity within the epithelial cells of the kidneys and bladder when compared to wild-type (Barbieri et al., 2014), due to multiple virulence factor modifications such as a reduced expression of type 1 pili and P fimbriae (Barbieri et al., 2014). In addition to this, the *fnr* regulatory homolog within *Salmonella* has shown evidence of positive virulence expression involved in cell flagella synthesis and motility during pathogenesis (Contreras et al., 1997; Fink et al., 2007). Therefore, *fnr* contributes to the pathogenesis of UPEC by regulating the expression of important virulence genes (Barbieri et al., 2014) in response to metabolism under different aerobic conditions.

#### Pst

The loss of the phosphate-specific transport system (Pst), encoded by the operon *pstSCAB-phoU*, results in the constitutive activation of the Pho regulon, involved in phosphate transport and metabolism. This constitutive activation has a negative effect on UPEC pathogenicity, with a Δ*pst* CFT073 mutant displaying a reduced fitness in a murine UTI model (Crépin et al., 2012). This effect on virulence is thought to be due to the decreased production of type 1 fimbriae in a Δ*pst* mutant, resulting in decreased invasion of bladder epithelial cells (Crépin et al., 2012), and demonstrates a link between nutrient sensing and UPEC pathogenicity.

## Linking niche-specific nutrients to the adaptation strategies of UPEC

There is now mounting evidence that the metabolic flexibility of the uropathogen UPEC is crucial for its ability to cause infection of the urinary tract. This is due to the advancements to understand the metabolome of human urine, resulting in the creation of the human urine metabolome database, and numerous studies that have detailed the effect of the loss of a single metabolic enzyme on the fitness of UPEC during urinary tract infection. Whilst this has provided valuable information about the metabolic response of UPEC during infection, understanding the complete metabolic requirements of UPEC in the urinary tract is currently challenging due to each metabolomics and genetic mutational studies being carried out in isolation. Thus, major conclusions correlating the levels of metabolites present in the host environment, and the role of the corresponding bacterial metabolic pathway involved in its catabolism are hard to draw. Ideally, whole-cell “omics” approaches, including transcriptomics and metabolomics, should be carried out in concert with single gene deletion studies to fully correlate the metabolic response with the virulence of UPEC during infection. Using such approaches will also minimize the variations due to sampling procedures, storage conditions and environmental influences. For example, analyzing the concentration of D-serine in urine, and correlating this to the role of *dsdA* (encoding D-serine deaminase) in fitness during UTI infection will allow us to further understand the direct contribution that amino acid utilization plays during UPEC metabolic adaptation.

New discoveries due to the advancement of “omics” technologies such as metabolomics are also shaping our understanding how UPEC interacts and adapts to nutritionally distinct environments as it moves through the gastrointestinal and urinary tracts. This data is imperative for the identification of new disease biomarkers to improve the diagnosis of UTIs (Mendrick and Schnackenberg, 2009) and for the discovery of novel antibiotic drug targets (Wishart, 2016). However, despite the potential power that metabolomics approaches have in resolving differences pertinent to disease processes (Lv et al., 2011), in comparison to other “omics” research (genomics and proteomics), metabolomics still struggles to cover even a small section of the entire metabolome, having identified <1% of the known human metabolome (Bouatra et al., 2013). Furthermore, due to the complexity of interconnectivity of metabolic processes, analysis of data obtained from metabolomics studies can prove to be difficult to interpret. Regardless, of these shortcomings, to date, metabolomics has successfully allowed comparison between UPEC and commensal *E. coli*, providing novel targets for virulence-based diagnosis (Su et al., 2016a). These virulence factors can be targeted to control the UPEC pathogenic mechanisms, thereby serving as potential drug targets (Hannan et al., 2012; Kostakioti et al., 2012; Subashchandrabose et al., 2014; Flores-Mireles et al., 2015; Yan et al., 2015; Su et al., 2016a,b).

## Metabolism as a potential antibacterial target for UTIs

### Drug targets

**Figure 3:**
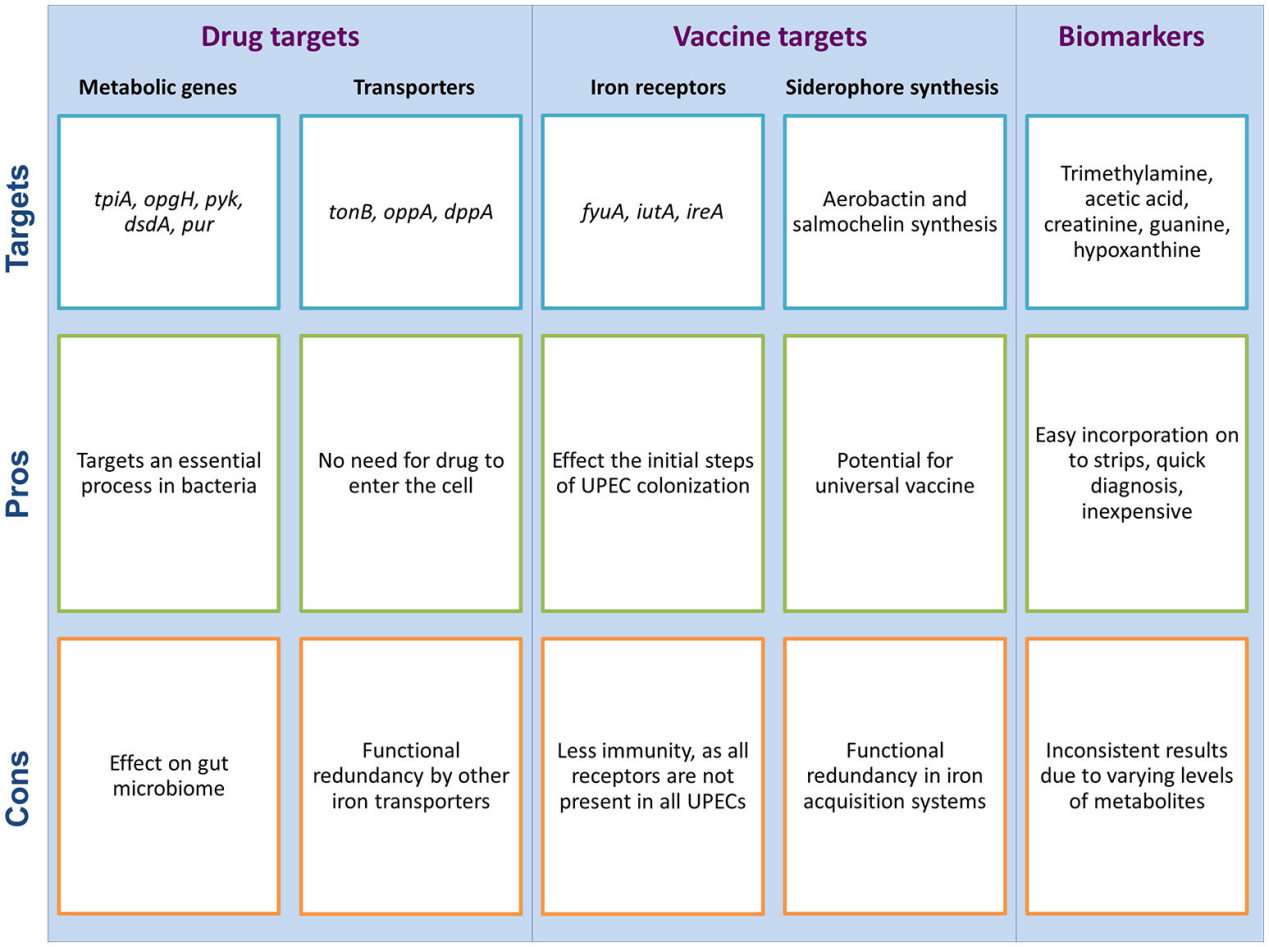
Metabolism as a potential target for the development of new drugs and vaccines for UTIs, and the use of urine as a diagnostic fluid for UTI detection; each with potential pros and cons associated with them. These potential targets are suggested by the authors as per the literature search.

Most of the presently used antimicrobial drugs target a limited number of bacterial processes, which are essential under nutrient-rich conditions. However, growth in a nutrient-rich medium in a laboratory setting does not reflect conditions within the host, wherein UPEC must co-exist with gut microbes for available nutrients, as well as display metabolic flexibility to adapt to the harsh and comparatively nutrient-scarce urinary tract environment. Under such circumstances, UPEC has developed novel strategies through metabolic adaptations in order to adjust and survive the challenging environment of the urinary tract. New drugs to be developed must target adaptive strategies of UPEC that are essential for survival in the host environment; for example, the metabolic pathways shown to be important for UPEC pathogenesis (Figure 2). Indeed, the longest standing antibiotic class of sulfonamides target the metabolic process of folate synthesis (Bourne, 2014), showing precedent for designing drugs to target bacterial metabolism. Investigation of metabolic pathways necessary for growth in a nutrient-restricted medium serve as a better proxy for infection-causing conditions inside the host, and has identified the essentiality of synthesis of amino acids, nucleotides and vitamins (Côté et al., 2016); therefore such processes are potential drug targets (Figure 3).

Under the nutrient stress conditions of the urinary tract, genetic and metabolic adaptation studies of UPEC has shown that central metabolic pathways are necessary for fitness of UPEC *in vivo*, and amino acids and peptides serve as the main source of carbon for UPEC during the urinary tract infection (Alteri et al., 2009). These pathways therefore form a critical component of the virulence of pathogenic microorganisms (Alteri et al., 2009, 2015; Lv et al., 2011, 2014; Yan et al., 2015), and serve as potential drug targets. For example, *tpiA*, which encodes triosephosphate isomerase involved in glycolysis, is important for kidney infection by UPEC (Alteri et al., 2009) and could therefore be a potential drug target for the treatment of pyelonephritis.

Urine, being nutritionally restrictive, is unable to provide all the growth factors required for bacterial growth, several of which are synthesized *de novo* by bacteria that are adapted to survival in this environment. Inhibitors of the synthesis process of these growth factors may therefore have therapeutic value for prophylaxis in the case of recurrent UTIs or for people with high risk factors for infection (e.g., during pregnancy), as well as treatment of uncomplicated UTIs (Hull and Hull, 1997). For instance, biosynthetic pathways involved in the synthesis of carbohydrates, purines and pyrimidines, and energy metabolism, those involved in D-serine utilization (*dsd* locus) and uptake of iron (*tonB*), are important factors contributing to bacterial fitness *in vivo*. Antibiotics that target these metabolic pathways or uptake systems hold the potential for treatment of UTIs (Vejborg et al., 2012).

### Vaccines

UTIs have a high recurrence rate of 25% (Bauckman and Mysorekar, 2016) and can occur in an individual for the duration of their lifetime, which requires long-term prophylaxis with antimicrobials (Kodner and Thomas Gupton, 2010; Kostakioti et al., 2012). Therefore, prevention using vaccines against uropathogens would be a promising alternative, especially for high-risk populations such as immunocompromised people and pregnant women (Brumbaugh et al., 2013).

Iron-acquisition strategies of UPECs are of interest for the development of novel diagnostics and therapeutics for UTIs (Miethke and Marahiel, 2007). The continuous struggle for iron between the host and the pathogen has spurred the iron-related coevolution of host and the pathogen as a defense and antidefense strategy by the two. This coevolution had resulted in the development of siderophore-neutralization strategies by the host in order to suppress the pathogen multiplication and reutilization strategies by the pathogen. Additionally, hosts have also developed cell conversion pathways and siderophore-based iron delivery systems to break the pathogen's supply of iron. Successful treatment strategies could therefore focus on weakening the iron-acquisition systems of UPECs or improving the siderophore-neutralization strategies of the host.

This is an area of interest for vaccine development not just restricted to UTIs, and has led to the development of vaccines targeting some iron acquisition proteins; like yersiniabactin uptake receptor FyuA, iron uptake aerobactin receptor IutA and iron-responsive siderophore receptor IreA (Brumbaugh et al., 2013), and siderophores yersiniabactin and aerobactin (Mike et al., 2016). However, targeting a single iron receptor is unlikely to yield a broad level of immunity because iron acquisition mechanisms show functional redundancy in *E. coli* (Garcia et al., 2011), and not all receptors are found in all UPEC strains (Brumbaugh et al., 2013). Therefore, an iron receptor-based multi-epitope vaccine against UPEC has the potential to provide broad-spectrum immunization, as has been developed for ExPEC, which has shown two-fold reduction in the bacterial load in a mouse peritonitis model (Wieser et al., 2010). It is possible that a similar multi-epitope vaccine would provide protection against diverse UPEC strains.

Siderophore synthesis is identified to modulate the differential metabolome of UPEC and non-UPEC strains, therefore the genes involved in synthesis of these siderophores are candidate targets for the development of drugs and vaccines against UPECs (Su et al., 2016a). Siderophores, which are traditionally considered an important virulence factor for UTI (Chaturvedi et al., 2014), have been recently demonstrated to enhance the virulence of UPEC partially by modulating their amino acid metabolism (Su et al., 2016a). Recent evidence has shown that the siderophores aerobactin and salmochelin present in UPEC have a stronger modulatory effect than other siderophores on amino acid metabolism (Su et al., 2016b). Therefore, siderophore biosynthesis and its associated metabolic pathways may be novel targets for therapeutics and virulence-based diagnoses.

### Diagnosis

The metabolic profile of infected urine, as analyzed by metabolomics, may reveal promising biomarkers to be used for the diagnosis of UTIs. Several examples have already been identified, including trimethylamine and acetic acid, which have potential for incorporation on to a dip strip (Karlsen and Dong, 2015). Another example is creatinine, a breakdown product of creatine, defined as a biomarker for the diagnosis of nephropathy (Ariza-Heredia et al., 2014). Higher creatine concentrations are observed in UPEC-grown urine in comparison to non-UPEC strain, therefore it may be used as a biomarker for the diagnosis of UPEC infection (Su et al., 2016a). Similarly, metabolites related to purine and pyrimidine metabolism are upregulated in UPECs as compared to non-UPECs, for example guanine and hypoxanthine (Su et al., 2016a). These two metabolites are known to be critical for the virulence of uropathogens (Vejborg et al., 2012; Karlsen and Dong, 2015), therefore hypoxanthine has the potential to be used as a biomarker for the diagnosis of UTIs. However, these potential markers for determining UTIs are from studies performed by culturing UPEC in urine obtained from healthy individuals under *in vitro* conditions (Karlsen and Dong, 2015), or by comparing the profile of metabolites produced by UPECs and non-UPECs under *in vitro* conditions (Su et al., 2016a), which are settings that are in the absence of host immune and cellular responses. The urinary composition of a healthy individual is highly likely to be very metabolically different from that of a UTI patient, partly due to the inflammatory response of the host. Furthermore, much of the work to establish the role of metabolism during UPEC infection has been performed using various mouse models, which raises concerns about the conservation of metabolic responses as compared to human infection. These factors together raises questions on using these identified metabolites as a means of determining UTIs. Thus, future studies should focus on understanding the metabolomic profile of urine from UPEC-infected individuals to provide reliable data for the identification of biomarkers of UTIs in response to the host immune response.

### Pros and cons of using metabolism as an antibacterial target

A number of studies have explored the urine metabolome of a healthy person and there are many examples comparing metabolite levels in a healthy person to a diseased person, thereby drawing conclusions about the role of specific metabolites in progression of a disease (Alonso et al., 2016; Feng et al., 2016). To our knowledge, none of the suggested metabolites from those studies have been standardized for use as a diagnostic marker for UTIs or developed as a potential drug target. This is most likely due to the complexity of metabolism itself, which makes it hard to get reliable and reproducible data required for any metabolite to be used as a disease marker, or any metabolic enzyme to be used as a drug target. Urine metabolomics analysis is usually done on pooled urine samples, as done in the Human urine metabolome project (Bouatra et al., 2013). This provides information about the metabolome of the combined urine sample obtained from different individuals rather than the urinary composition of one individual. Therefore, the levels of metabolites obtained from such studies do not hold a true potential to be standardized for the determination of UTIs. Further complicating is the fact that the levels of metabolites can vary merely due to different physiological conditions of a person which is unrelated to disease status (discussed in the next paragraph). Therefore, the parameters for threshold levels of metabolites must be carefully considered before determining their potential for UTI diagnosis.

Although there are a number of potential biomarkers identified through individual studies, one very critical point to consider before selecting such metabolites as a suitable UTI biomarker is that the concentration of certain metabolite can naturally vary by as much as seven-fold in different individuals, depending on a number of factors like age, gender, health status, diet and activity level (Saude et al., 2007; Bouatra et al., 2013). Overall, dietary intake appears to be the most influential factor on the concentrations of urinary metabolites, with the type of diet dictating the metabolite concentration. For example, mannitol, which is commonly present in foods like carrots, apples, pineapples and asparagus, is commonly found in human urine because of its poor absorption by the body (Bouatra et al., 2013). Similarly, chocolate and meat intake are known to increase the concentrations of polyphenol-derived phenolic acids and 1-methylhistidine, respectively, in the urine (Sjölin et al., 1987; Rios et al., 2003). Some gender-related effects are shown to influence the level of creatinine, a major component of the human urine and often used to normalize metabolite concentrations of urine samples. Increased intake of creatine or a protein-rich diet is also shown to increase the urinary excretion of creatinine (Taylor et al., 1989). In addition to dietary influence on the urine metabolome, the levels of metabolites in urine vary with the physiological status; for example, the levels of 3-hydroxybutyric acid increases during fasting (Bouatra et al., 2013). Therefore, a careful consideration of patient gender, diet and physiological status is necessary before drawing conclusions about any potential UTI diagnostic biomarker. Moreover, further studies need to be conducted to investigate the contribution of each of these factors to the urine metabolome. However, such studies would require a significant amount of technical expertise, considering the diversity of urinary metabolites and the range of factors contributing toward the variation in levels of those metabolites. In addition to exploring the contribution of such factors to the urine metabolome, conditions will need to be standardized for the collection of samples for examination. For example, baseline concentrations of the biomarker may vary if the urine sample is collected under fasting conditions as compared to non-fasting conditions, which has implications in the diagnosis of a UTI.

One important point to consider when developing drugs that target the process of metabolism, or for that matter, for all drugs that target essential bacterial processes, is the potential side effects on the balance of the microbiome in the gut. Therefore, ideally, the target to be selected for drug development should be specific to UPEC and should have minimal effect on the gut microbiota. Since the availability of iron to UPEC is limiting in the urinary tract, the uptake of iron using *tonB* transporter is important for UPEC virulence. Therefore, the iron transporters in UPEC are potential drug targets. However, for this target to be practically viable, further research needs to be done toward investigating the role of iron transporters for intestinal survival of the members of gut microbiome, as the potential drug will have consequences on the gut microbiome as well.

Despite potential shortcomings with using metabolomics as an antibacterial target, the essentiality of metabolism for bacterial survival and the requirement of its adaptation inside the urinary tract make it a promising target for UPEC treatment. However, the current knowledge of the urine metabolome is incomplete, but further advancements in techniques to fully quantify metabolites and their levels in urine will aid our understanding of the human urine metabolome. In concert, studies are being continuously done to explore the role of metabolic enzymes in UPEC adaptation inside the urinary tract and new metabolic enzymes are being discovered. Identification of these metabolic enzymes and the roles they play in UPEC adaptation, along with correlating those adaptation strategies to the levels of corresponding metabolites in both the bacterial cell and human urine will allow us to better identify novel drug targets aimed specifically at UPEC metabolism.

## Conclusion

UTIs are one of the most prevalent bacterial infections, resulting in billions of dollars of expenditure every year (Stamm and Norrby, 2001). Of greater concern however is the increasing issue of antibiotic resistance amongst pathogens causing UTIs (Flores-Mireles et al., 2015). Metabolism is an essential bacterial process and has been shown to be critical for UPEC adaptation to specific host environments during infection, making it an ideal potential treatment target. Further, identifying the metabolic adaptation strategies of UPEC strains can help uncover an entirely new area with prospective exploitation for the development of novel antimicrobials to treat UTIs.

Our current understanding of the urine metabolome and UPEC metabolic adaptation is incomplete, partially due to technological shortcomings and the complexity and quantity of metabolites produced by UPEC, especially in response to interaction with the host environment. However, there have been recent efforts to understand the contribution of metabolism to UPEC virulence. From the current knowledge, limited availability of iron in urine appears to be the growth-limiting factor for UPEC growth in human urine; therefore UPEC systems involved in the uptake and utilization of iron seem to be a very promising target for novel drug development. The most abundant amino acid known to be present in urine is D-serine, which has been shown to be important for the virulence of UPEC (Vejborg et al., 2012). Therefore, the systems involved in catabolism of this amino acid also can be potential drug targets. Further advancements in “omics” techniques, along with a multi-omics approach, will provide better understanding of the adaptation strategies of UPECs and will guide us in future treatment developments.

## Author contributions

RM and AB conceived the manuscript. RM, DM, ID, and AB wrote the manuscript. ID, EH, and AB edited the manuscript. All authors provided comments during the preparation of the manuscript.

### Conflict of interest statement

The authors declare that the research was conducted in the absence of any commercial or financial relationships that could be construed as a potential conflict of interest.
